# Band Gap Renormalization at Different Symmetry Points in Perovskites

**DOI:** 10.1021/acsphotonics.4c00082

**Published:** 2024-05-17

**Authors:** Lijie Wang, Razan Nughays, Jun Yin, Chun-Hua Shih, Tzung-Fang Guo, Omar F. Mohammed, Majed Chergui

**Affiliations:** † Lausanne Centre for Ultrafast Science (LACUS), ISIC, École Polytechnique Fédérale de Lausanne (EPFL), CH-1015 Lausanne, Switzerland; ‡ Advanced Membranes and Porous Materials Center (AMPM), Division of Physical Science and Engineering, King Abdullah University of Science and Technology (KAUST), Thuwal 23955-6900, Kingdom of Saudi Arabia; § Department of Applied Physics, The Hong Kong Polytechnic University, Kowloon 999077, Hong Kong, P. R. China; ∥ Department of Photonics, National Cheng Kung University, Tainan 701, Taiwan; ⊥ KAUST Catalysis Center, Division of Physical Sciences and Engineering, King Abdullah University of Science and Technology (KAUST), Thuwal 23955-6900, Kingdom of Saudi Arabia; # Elettra Sincrotrone Trieste, Strada Statale 14 - km 163,5, 34149 Basovizza, Trieste, Italy

**Keywords:** broad-band UV transient absorption, ultrafast spectral response, photoexcitation, global lifetime analysis, many-body interactions

## Abstract

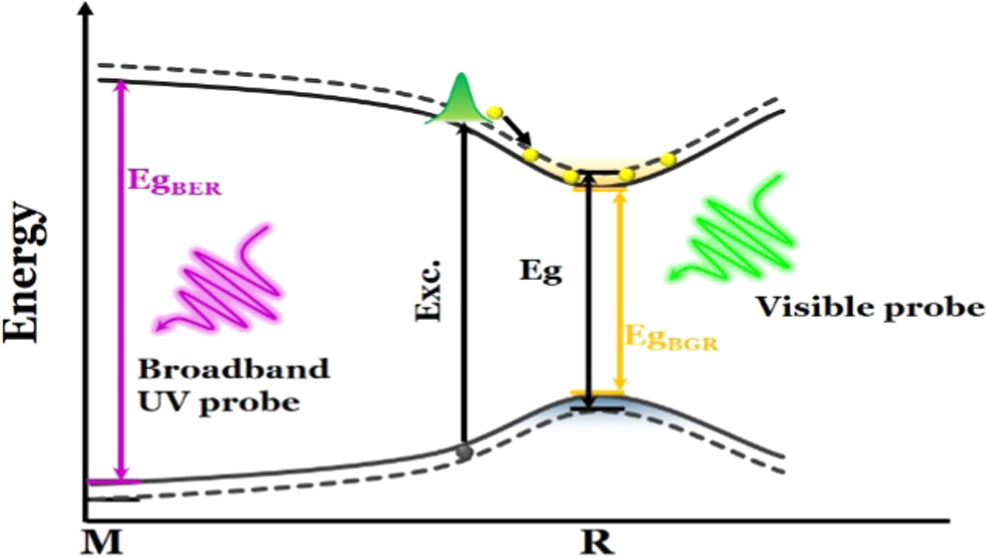

Using ultrafast broad-band transient absorption (TA) spectroscopy of photoexcited MAPbBr_3_ thin films with probe continua in the visible and the mid- to deep-ultraviolet (UV) ranges, we capture the ultrafast renormalization at the fundamental gap at the *R* symmetry point of the Brillouin zone (BZ) and a higher energy gap at the *M* symmetry point. Advanced global lifetime analysis and lifetime density distribution analysis are applied to extract quantitative information. Our work confirms the similarity of the response at both high-symmetry points, which indicates a band edge renormalization that rises within the instrument response function (IRF, ∼250 fs) and decays in ca. 400–600 fs, undergoing an energy red shift of 90–150 meV. The reported time scale corresponds to the decay of free carriers into neutral excitons. The ability to monitor different high-symmetry points in photoexcited perovskites opens exciting prospects for the characterization of a large class of materials and for photonic applications.

## Introduction

Semiconductors are known to exhibit large changes in their optical properties in the region of the fundamental band gap upon photoexcitation.^1−3^ These changes are due to the creation of electron–hole pairs that modify the complex optical dielectric function. This is manifested by various concurrent processes, which involve single-particle states (namely, phase-space filling) and those that can be attributed to many-body interactions among the doped carriers (i.e., long-range Coulomb screening and band gap renormalization). Phase-space filling originates from the Pauli exclusion principle, which applies to the electrons and holes constituting the excitons. As a consequence, this generates a finite exclusion volume in phase space for each exciton, decreasing the valence band (VB) to conduction band (CB) transition probability, which translates into a reduction of the oscillator strength of the excitonic transition in the optical spectra. Many-body interactions affect the underlying electronic states and, thus, alter the exciton energy and composition. This occurs via the direct Coulomb interactions of electrons and holes as well as the exchange Coulomb interaction, particularly evident at high charge carrier densities. On the one hand, long-range free carrier screening modifies the exciton Coulomb potential. On the other hand, the excess electrons also lead to band gap renormalization (BGR), i.e., a reduction of the fundamental quasiparticle gap. Thus, the electronic screening leads to the simultaneous renormalization of both the exciton and the electronic gap (Figure S1).

BGR is one of the first events that occurs upon photoexcitation. Understanding it holds great importance for applications in photonics, ultrafast optical switching, and even unconventional superconductivity.^1,4−8^ BGR concerns the fundamental absorption band edge, which in most semiconductors lies typically in the visible spectral region.^9−12^ However, a more complete characterization of materials properties and response to photoexcitation implies probing the band edge renormalization (BER) at transitions higher than the fundamental gap. These originate either from higher (deeper) CB/VB or from band gaps at different high-symmetry points of the lowest CB in the band structure (BS) diagram. This aspect has, to our knowledge, barely been investigated in the literature.^2^ It requires an approach that can monitor a wide wave-vector space of the BS diagram of the material. Angle-resolved photoelectron spectroscopy (ARPES) at extreme ultraviolet energies is ideal in this respect,^13^ but in the absence of chemical doping^14,15^ or photodoping,^16,17^ it monitors only the VB structure. In addition, even in the case of chemical doping or photodoping, only the lowest valley(s) of the CB that contain(s) electrons can be visualized. It is, however, seldom the case that more than the lowest CB minimum is populated. Using photodoping and time-resolved ARPES,^18,19^ Roth et al.^16^ investigated photoexcited black phosphorus and reported the temporal evolution of the CB and VB at the Γ point of the BS diagram of the material. However, for estimating the BGR energy shift, they focused on only the VB maximum. An alternative method that can resolve in both energy and momentum space fundamental excitations in solids is resonant inelastic X-ray scattering (RIXS),^20^ which is starting to be implemented in the time domain^21,22^ but still awaits full implementation.

An alternative approach is to perform transient absorption or reflectivity (TA/TR) studies using (a) broad-band continuum probe(s) that would embrace an as wide as possible observation window in energy space. Of course, this approach does not provide momentum (or wave vector) resolution as ARPES or RIXS does, but with the help of the BS diagram, one can make contact between the momentum (wave vector) and energy spaces. This approach was implemented in the case of the indirect band gap (BG) material, anatase TiO_2_, using a broad-band mid- to deep-ultraviolet (UV) continuum probe,^15^ reporting transitions in different regions of the BS diagram, though the gap renormalization was not specifically addressed in this work. The TA approach provides the additional advantage of canceling out scattering effects by taking the difference between pumped and unpumped sample absorption spectrum,^15,23^ and since it is a derivative-based method, it can reveal barely resolved features of the linear absorption spectrum. These two last aspects are of importance, as the absorption spectra of semiconductors are often quasi-continuous beyond the optical gap. Here, we implemented TA with broad-band continual in the case of one of the most promising optoelectronic materials, the MAPbBr_3_ perovskite.

The choice of MAPbBr_3_ is motivated by its potential applications in solar energy research,^24^ as detectors for high-energy radiation^25^ and for optoelectronic devices.^26,27^ It is and has been richly investigated, especially at the fundamental BG in the visible spectral range, and BGR is also well documented.^7,12,28−32^ Its absorption spectrum shows modulations above the fundamental band gap (VB1 → CB1, ∼ 2.3 eV labeled 1), which correspond to at least three edges representing transitions from the upper VB at the M and *X* high-symmetry points to the lowest CB (labeled 3 and 4: VB1 → CB1, ∼3.8 eV and VB1 → CB1, ∼4.5 eV), or from a VB sub-band to the lowest CB at the *R* point (labeled 2: VB3 → CB1 at ∼3.4 eV), based on the BS diagram.^33,34^ Here, we excite the material with a low pump fluence pulse at 3.1 eV, i.e., well above the bang gap at 2.3 eV, which implies that we generate free carriers. These will induce a BGR that survives as long as free carriers decay to form neutral excitons. By comparing the BGR with the renormalization at band edges of different high-symmetry points (hereafter called band edge renormalization, BER), we can quantify the way photoexcited charge carriers affect the system in different regions of the Brillouin zone (BZ). In the present case, we deal with the fundamental gap at the *R* point and the band edge at the *M* point of the BZ, under identical excitation conditions. It is important to stress that as the *M*-point lies well above the excitation energy, its response is purely due to edge renormalization and is not contaminated by the presence of charge carriers, yielding effects such as Pauli blocking.

To analyze the early time photoinduced data, we employed a global lifetime analysis (GLA) method (see the Supporting Information for the method), which yields decay-associated spectra (DAS),^35,36^ and we determined, respectively, the lifetimes and energy red shift of BER in MAPbBR_3_ material to be in the range of ca. 400–600 fs, and of ∼120 meV at the *R* and *M* points, respectively. The time scale of the decay of the BGR/BER represents the formation time of neutral excitonic particles in the material. These results confirm that the photoinduced transient gap reduction occurs at different high-symmetry points, exhibiting nearly identical behaviors for the lowest CB.

## Experimental Section

### Sample Preparation and Characterization

For MAPbBr_3_ films, prior to deposition, the quartz substrates underwent a sequential cleaning process. First, the substrates were subject to ultrasonic treatment in a detergent, deionized water, acetone, and isopropyl alcohol. After drying, the cleaned substrates were further treated with UV ozone (Model: 42, Jelight) for 25 min. The MAPbBr_3_ precursor solution was prepared by combining 0.015 g of MABr (Dyesol) with 0.0481 g of PbBr_2_ (Sigma-Aldrich, 99.999%) in an anhydrous dimethyl sulfoxide (DMSO, Sigma-Aldrich) solution (1070 μL) at 60 °C. The solution was stirred for 12 h. To fabricate the MAPbBr_3_ perovskite thin film, a consecutive two-step spin coating process was employed. The solution was spin-coated onto the quartz substrates at 500 rpm for 7 s, followed by spin coating at 4000 rpm for 70 s. Additionally, at 43 s during the spin coating, 250 μL of chloroform solvent was dropped onto the surface of the precursor film. Subsequently, the MAPbBr_3_ perovskite film was annealed on a hot plate at 70 °C for 10 min. It is noteworthy that all of the procedures for preparing the MAPbBr_3_ precursor solution and films were conducted inside a nitrogen-filled glovebox with oxygen and moisture levels maintained below 1 ppm.

For MAPbBr_3_ single crystals used for ellipsometric experiments, the precursor MABr (0.748 g) was dissolved in anhydrous dimethylformamide (4 mL) in a 20 mL glass vial to form a clear solution. Then, PbBr_2_ (2.452 g) was added into the glass vial with stirring to obtain a nearly saturated clear MAPbBr_3_ solution. The glass vial was then placed onto a hot plate at 50 °C without disturbance for slow evaporation. Bulk MAPbBr_3_ single crystals with dimensions in the centimeter range were obtained from the solution after 12 h. These procedures were all performed inside a fume hood.

### Transient Spectroscopic Measurements

The experiments were performed under 3.1 eV pump photon energy using two different setups: one for a broad-band visible probe and the other for a broad-band deep-UV probe.

(a) For the broad-band visible probe setup, a 1 kHz regenerative amplifier provides 30 fs pulses at 800 nm with an energy of approximately 720 μJ per pulse. A noncollinear optical parametric amplifier (NOPA) was utilized to generate tunable visible pump pulses with ∼15 nm bandwidth and energy ranging from 2 to 4 μJ per pulse. The probe beam was focused onto a CaF_2_ plane to generate white light in the range of 450–750 nm (1.65–2.75 eV).
(b) For the broad-band deep-UV probe setup,^37,38^ a 20 kHz Ti/sapphire regenerative amplifier (KMLabs, Wyvern500) provides 50 fs pulses at 800 nm with an energy of 0.6 mJ. These pulses were used to pump a NOPA, generating sub-90 fs visible pulses at 13 μJ per pulse in the range of 510–740 nm (1.68–2.43 eV). About 40% of the NOPA output was used to generate broad-band UV probe pulses with a bandwidth of ∼100 nm through an achromatic doubling scheme.^39^ The probe pulses were further compressed using chirp mirrors and were determined with a commercial FROG system (Swamp Optics) to be <20 fs pulse duration. The relative polarization between the pump and probe beams was set at the magic angle (54.74°) using a half-wave plate to avoid photoselection effects. After passing through the sample, the transmitted broad band probe beam was focused into a 5 m multimode optical fiber, which was coupled to the entrance slit of a 0.25 m imaging spectrograph (Chromex 250is). The beam was dispersed by a 150 g/mm holographic grating and imaged onto a multichannel detector consisting of a 512-pixel CMOS linear sensor (Hamamatsu S11105) with a pixel size of 12.5 × 250 μm^2^. The pixel readout rate could reach up to 50 MHz. The typical spot sizes of the pump and probe beams were approximately 120 and 50 μm full widths at half-maximum, respectively.

In all measurements, we used a very low pump fluence, at 400 nm (3.1 eV), of approximately 50 μJ/cm^2^ with ∼10% uncertainty due to the laser power measurement and laser beam spot size. The pump power was recorded on a shot-to-shot basis using a calibrated photodiode for each pump wavelength, enabling the normalization of the data for the pump power. The instrument response function (IRF) was determined by measuring the cross-phase modulation (CPM) signal at time zero of the pure quartz substrate, and was found to be ∼250 fs. The thin-film perovskite samples were mounted in a film sample holder with a nitrogen gas flow to protect the sample surface. The probe signal was measured after transmission through the sample, and its detection was synchronized with the laser repetition rate.

### Spectroscopic Ellipsometry

Spectroscopic ellipsometry was performed using an M-2000 DI device (J. A. Woollam), which operated in the 193–1690 nm wavelength range. The sample was measured at a minimum of three angles of incidence (65, 70, and 75°), and the data analysis was performed using the Complete EASE 6.51 software package to generate the absorption coefficient of the perovskite signal crystal.

### Band Structure Calculations

We performed the BS calculations using the projector-augmented wave method implemented in the Vienna Ab initio Simulation Package code.^40,41^ The GGA and PBE exchange-correlation functionals were used, and van der Waals interactions were also included in the calculations using the zero-damping DFT-D3 method of Grimme. A uniform grid of 6 × 6 × 6 *k*-mesh in the Brillouin zone was employed to optimize the crystal structure of cubic-phase MAPbBr_3_. The energy cutoffs of the wave functions were set to 500 eV for bulk MAPbBr_3_.

### Global Analysis

We conducted a GLA that simultaneously examines multiple kinetic traces recorded at different probe energies, using a discrete sum-of-exponentials function^35^
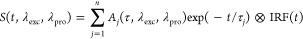
1where τ represents the global lifetimes and *A* is the amplitude for each kinetic trace. The detected signals are convoluted with the IRF, which is modeled by a polynomial function^36^
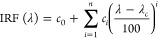
2The time zero position at the central wavelength, λ_*c*_, is given by *c*_0_. GLA yields the so-called DAS, where the pre-exponential amplitudes for each lifetime component are plotted against the probe wavelength, λ_pro_, and the DAS is a compact representation of the kinetic information in the data. Figure 2c presents the TA map retrieved from the extracted DAS, which successfully captures all of the spectral features observed in Figure 2a.

### Lifetime Density Distribution Analysis

To determine the lifetime of the BER effect at ∼3.8 eV, we performed lifetime density distribution (LDD) analysis that compresses the kinetic information into a distribution map (see the SI for the method). The great number of spatial, energetic, and temporal degrees of freedom of the ultrafast responses and their matrix produces a continuous distribution of individual exponential decays^35^
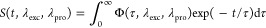
3The function *S*(*t*) represents the Laplace transform of the spectral distribution function, Φ(τ).^42^ The integral in eq 3 needs to be discretized into a quasi-continuous sum of *n* exponential functions similar to eq 1 but with *n* typically >50. Thus, the LDD analysis offers a comprehensive overview of the kinetics, allowing for the resolution of complex analysis issues, such as nonexponential or stretched exponential kinetics.^35^

## Results and Discussion

**Figure 1:**
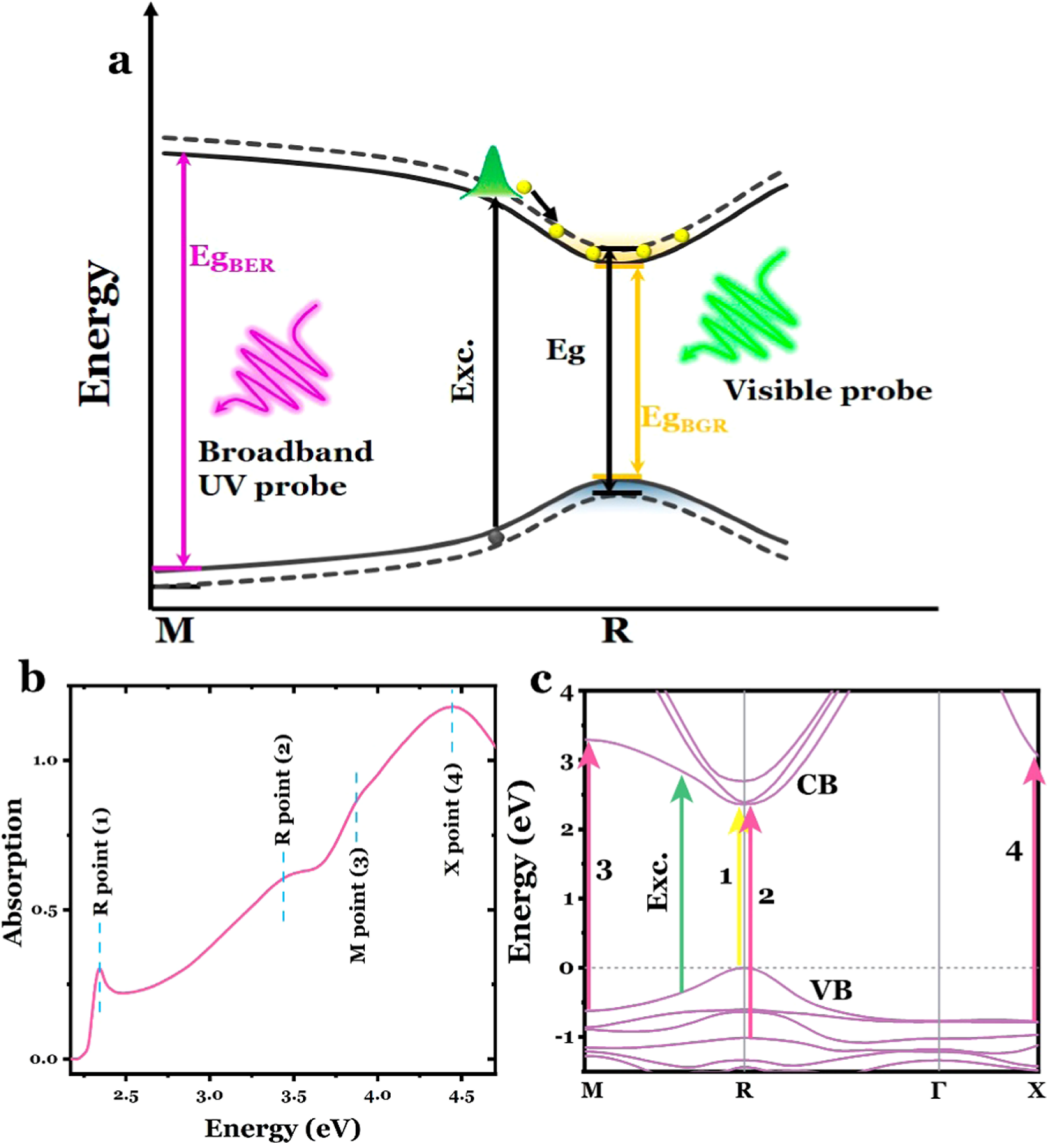
(a) Schematic diagram of the visible and deep-UV-based detection of band transitions in the MAPbBr_3_ perovskite material. (b) Absorption spectrum of a MAPbBr_3_ single crystal. The absorption peaks at each energy are labeled according to their corresponding interband transitions at different symmetry points. (c) Calculated energy band diagram. The 3.1 eV excitation is indicated by the green arrow, while the probed bleached signals that can be detected by the employed broad-band visible and mid- to deep-UV probes are represented by the yellow and red arrows, respectively. The numbers 1, 2, and 3 are labeled in the order of the CB and VB at each symmetry point.

Figure 1a shows a schematic diagram depicting the detection of different band transitions in the MAPbBr_3_ perovskite material by using both visible and deep-UV probes. Upon absorption of photons at the pump photon energy (3.10 eV), a nonequilibrium carrier population is generated, leading to a reduction of the energy gap on a fs time scale. The BGR at the *R* point (corresponding to the fundamental BG) is detected using a visible probe, while the high-energy interband transition at the *M* point (labeled 3 in Figure 1b, at ∼3.8 eV, and in the BS diagram in Figure 1c) can only be detected using a broad-band UV probe. These transitions are annotated in the absorption spectrum that was derived from spectroscopic ellipsometry measurements on a single crystal (Figure 1b), and they are identified with the help of the calculated band structure diagram in Figure 1c. Specifically, transitions 1 and 3 at the *R* and *M* points, respectively, arise from VB1 → CB1 transitions.^43^ The signature of BGR in a transient spectrum is a derivative-like line shape with a positive low-energy side and a negative high-energy side (Figure S1). It is worth noting that as our continuum probe covers the 3.3–4.3 eV range, this hinders the detection of BER at transitions 2 and 4, and we therefore focus our study on transition 3 for the higher-energy edge.

**Figure 2:**
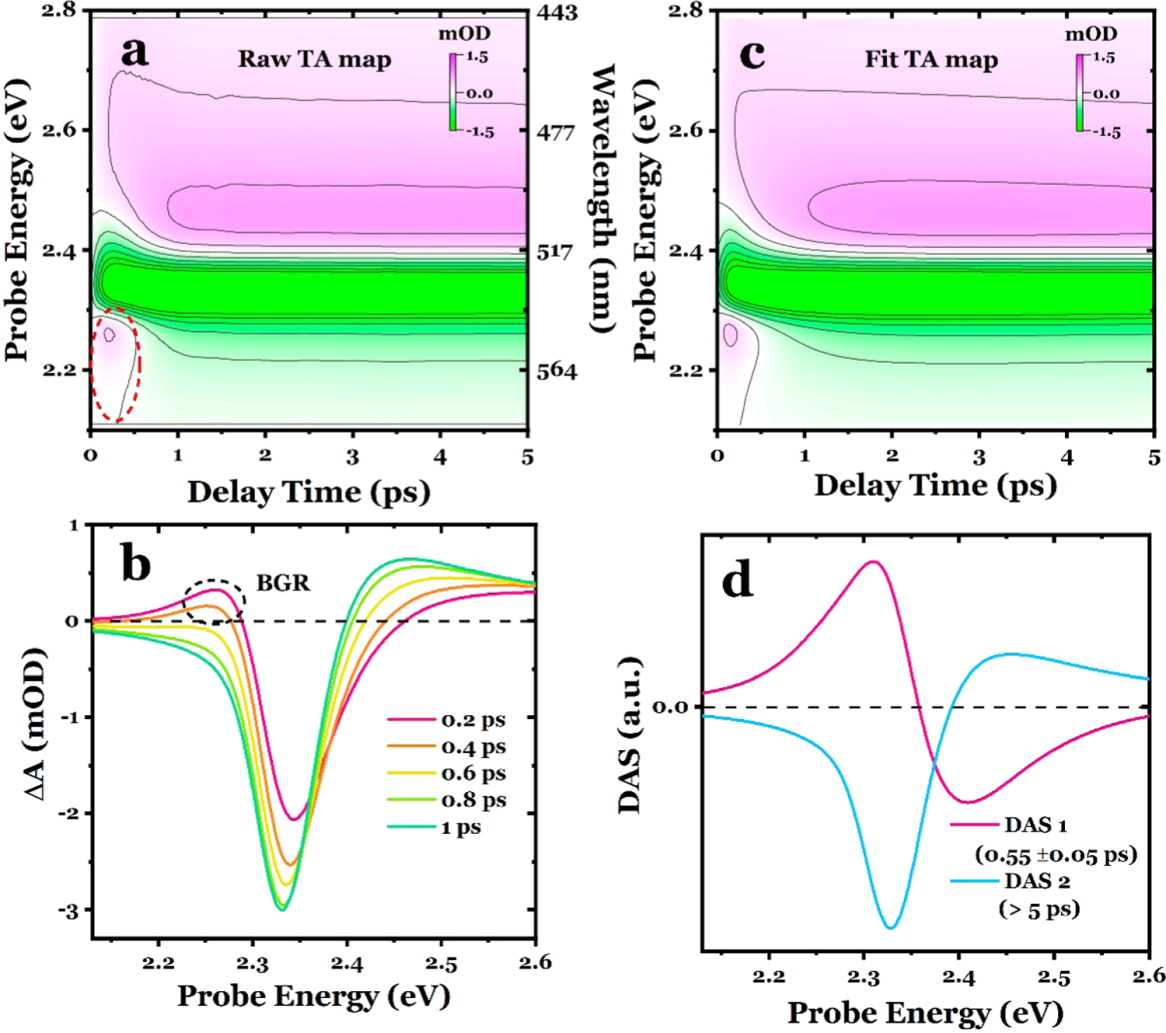
(a) Experimental TA time-energy map probed in the visible spectral region. (b) Photoinduced transients at 0.2, 0.4, 0.6, 0.8, and 1 ps. (c) Fitted TA map, by using global lifetime analysis method. (d) Decay-associated spectra corresponding to fast and slow evolution processes. The fitting was performed within a time window of 5 ps.

We used the visible spectral region, which covers the VB1 → CB1 transition at the *R* point (fundamental BG transition), as previously studied.^7,32,30^Figure 2a shows the time-energy TA map in a 0–5 ps time window of the MAPbBr_3_ perovskite excited at 3.10 eV, and Figure 2b shows the corresponding spectral traces at various delay times, revealing distinct negative and positive features from low to high energies along with a small positive shoulder on the lower-energy side at 0.2 and 0.4 ps. These figures display the characteristic bleach at the optical BG transition at ∼2.35 eV (see Figure 1b), mainly due to phase-space filling,^2^ accompanied by a broad, weak absorption signal on the higher-energy side, due to Coulomb screening.^1,3,44^ These features are consistent with previous reports on similar perovskite systems.^7,32,45,46^ In addition, a small positive signal promptly emerges on the lower-energy side of the BG (indicated by the dashed red circle in Figure 2a), which we attribute to BGR caused by the presence of hot carriers on time scales of tens to hundreds of femtoseconds.^12^ As the excited hot carriers occupy fewer states at the new, lowered band edge compared to colder-carrier distributions, the TA signal at the renormalized band edge displays a photoinduced absorption (PIA), which disappears on a sub-ps time scale as the carriers rapidly cool and form neutral excitons.^12^

Next, we performed a GLA that simultaneously examines multiple kinetic traces recorded at different probe energies (see the SI for the method). Figure 2c presents the TA map retrieved from the extracted DASs, which successfully captures all of the spectral features observed in Figure 2a. The resulting DASs associated with two different lifetimes are depicted in Figure 2d and correspond to an initial sub-ps process (DAS1, 0.55 ± 0.05 ps) and a long-term (>5 ps, see Table S1 for the time parameters) evolution process (DAS2). DAS1 exhibits a derivative-like shape, with positive values on the low-energy side and negative ones on the high-energy side, as expected for the BGR and its immediate red shift after photoexcitation. On the other hand, DAS2 shows the shape of the photoinduced electronic signal at a longer time scale (Figure 2b).

**Figure 3:**
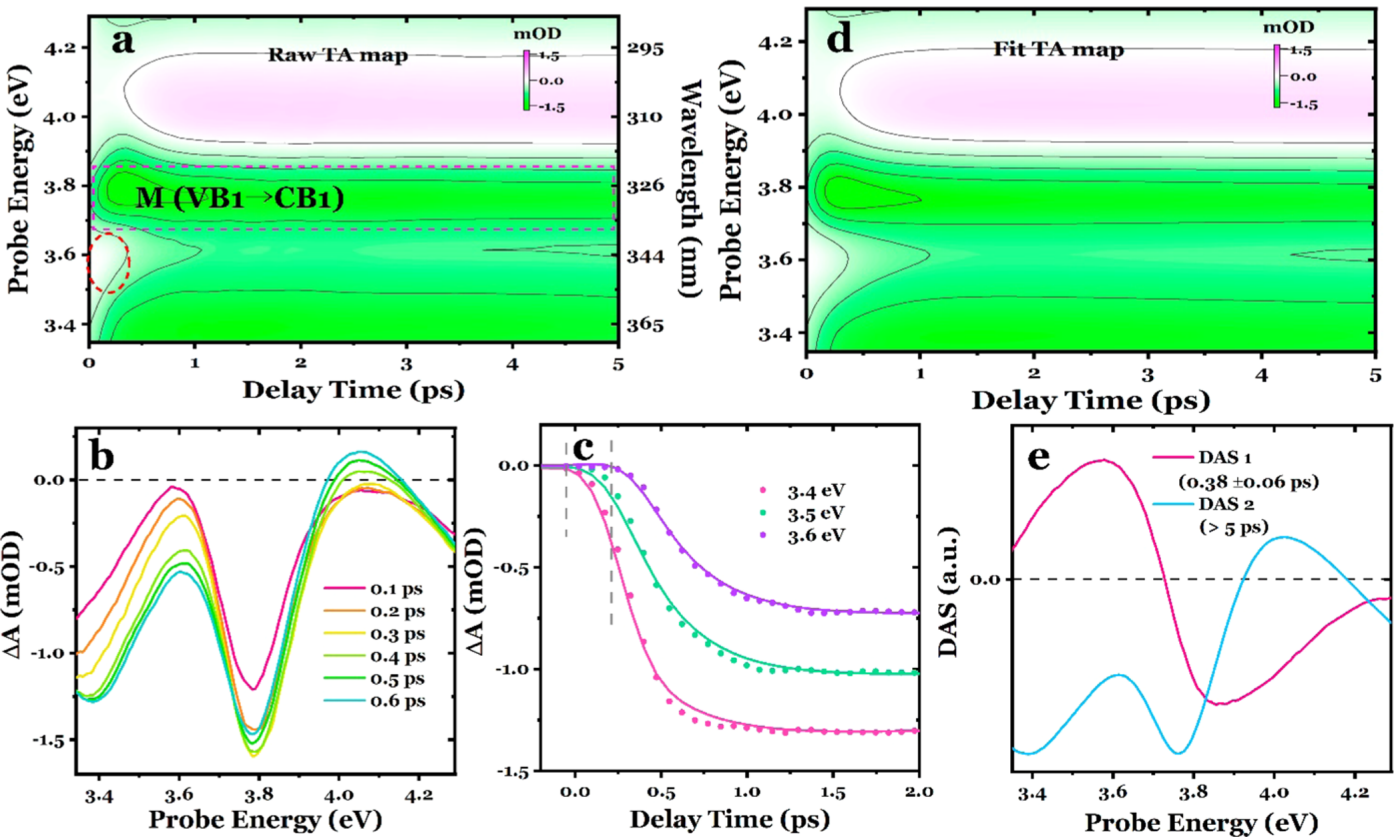
(a) Experimental TA time-energy map probed in the mid- to deep-UV spectral region. (b) Photoinduced transients probed in the UV spectral region, at 0.1, 0.2, 0.3, 0.4, 0.5, and 0.6 ps, respectively. (c) Early temporal traces probed at 3.4, 3.5, and 3.6 eV, along with their fits. (d) Fitted TA map, using parameters from the global fit (lifetime) analysis. (e) Decay-associated spectra corresponding to a fast and a slow process after photoexcitation.

We now turn to the band edge response at the *M*-point. Figure 3a presents the time-energy TA map in the 3.3–4.3 eV region under identical pump conditions as for the experiments in the visible range. Additionally, Figure 3b displays the corresponding spectral traces obtained from UV-probed measurements. The transients exhibit three pronounced negative bands at approximately 3.4, 3.8, and above 4.3 eV, accompanied by a positive signal around 4.15 eV. The assignment of these features was given in the Introduction section and is shown in Figure 1b,c.

Notably, there is a weak positive signal around 3.6 eV, which is close to zero and disappears within approximately 0.5 ps (indicated by the dashed red circle in Figure 3a). This behavior is also visible in Figure 3b. Unlike the immediate negative response observed at 3.4 and 3.8 eV after photoexcitation (e.g., at 100 fs), the signal at 3.6 eV remains close to zero before the negative component increases sharply. This delayed increase in negative amplitude is particularly evident in the early temporal traces shown in Figure 3c. In contrast to the TA signal at 3.4 and 3.5 eV, the 3.6 eV signal exhibits a delayed signal increase of ∼0.3 ps. Additionally, this short-lived signal appears slightly below the 3.8 eV band, which corresponds to the energy gap transition at the *M* symmetry point in the BZ (the early time traces at ∼3.8 and 4.15 eV are also compared in Figure S2). However, the strong superposition of negative signals may obscure the nature of the positive response at around 3.6 eV. Nevertheless, to unravel the underlying spectral components at different delay stages, a GLA was performed to generate the DAS that provides the spectral components at each lifetime. Figure 3d presents the reconstructed TA map obtained from GLA, and Figure 3e shows the resulting DAS1 and DAS2. The DAS2 basically resembles the long-term spectral responses. DAS1, representing the spectral amplitude of the subpicosecond (0.38 ± 0.06 ps) lifetime component, exhibits a similar BGR derivative-like profile as in the visible region, suggesting a BER at the M symmetry point (see Figure 1 and Note S2, SI). The DAS lifetimes in the visible and UV spectral regions are listed in Table S1. Since the excitation photon energy is lower than the energy gap at the *M* point (∼3.8 eV), the response is solely due to a gap renormalization and cannot be contaminated by charge carriers present at this point, as these charge carriers are energy-wise generated below the edge we are monitoring. The effect at this symmetry point is therefore only due to BER.

**Figure 4:**
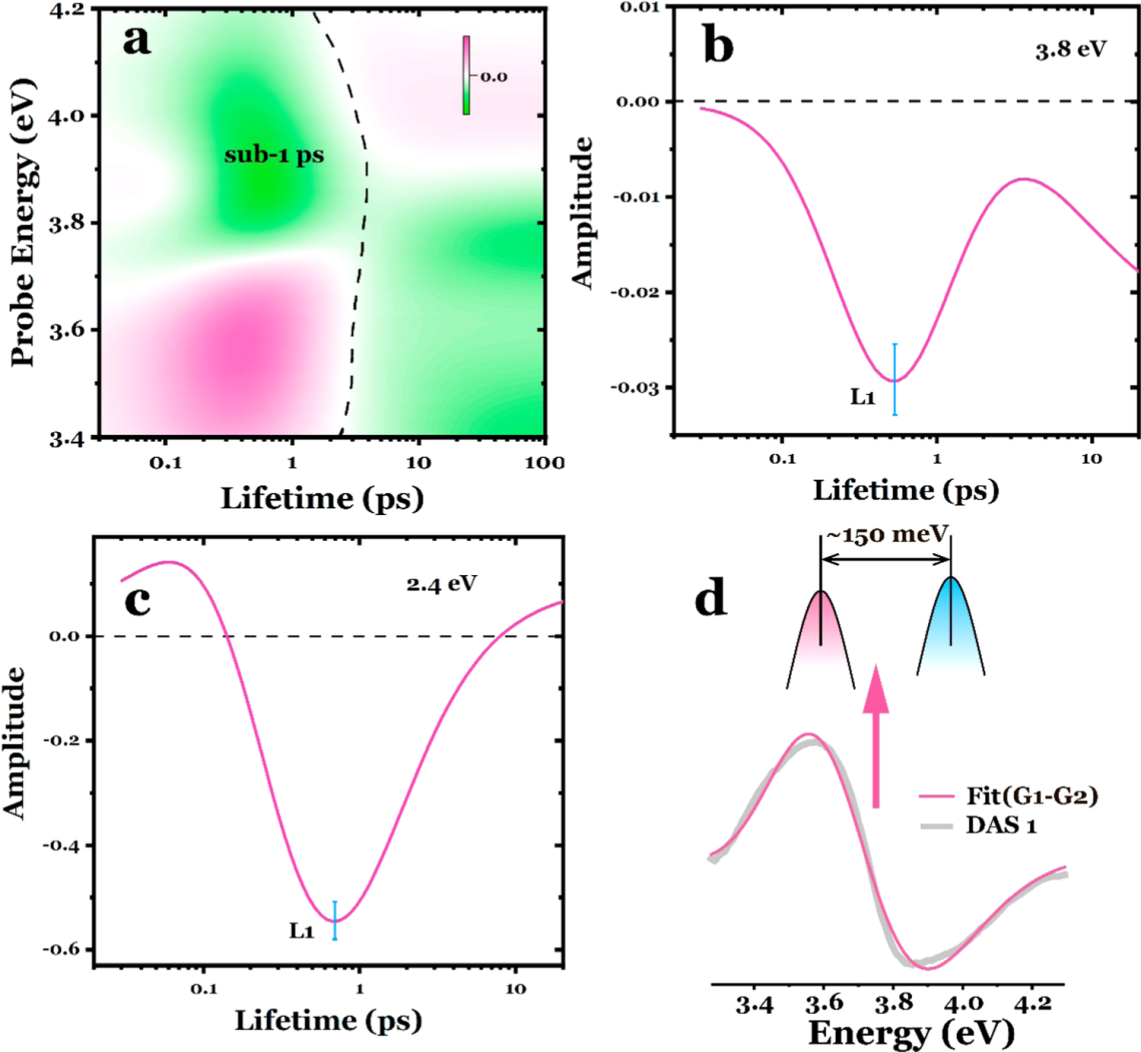
(a) Lifetime density distribution map within 100 ps, fitted to the Δ*A* data in Figure 3a. The spectral amplitudes observed at shorter lifetimes (e.g., <1 ps) exhibit a similar profile to DAS1, while the amplitudes at longer lifetimes (e.g., 10–100 ps) bear resemblance to DAS2. (b) Lifetime trace of the LDD at the probe energy of 3.8 eV, a clear peak (L1) can be resolved in the first 1 ps, with the L1 at the amplitude maximum position of ∼530 ± 106 fs. (c) Lifetime trace of the LDD at the probe energy of 2.4 eV. (d) Schematic diagram illustrating band energy reduction (red shift) and fitting of the DAS1 signal using two Gaussian functions (Gaussian1 minus Gaussian2). A band red shift of approximately 150 meV was obtained.

To determine the lifetime of the BER at ∼3.8 eV, we performed a lifetime density distribution (LDD) analysis that compresses the kinetic information into a distribution map (see the SI for the method). Figure 4a presents the LDD map, which extends up to a lifetime of 100 ps and is fitted to the Δ*A* data depicted in Figure 3a. Gaussian lineshapes were used to simulate a three-dimensional lifetime distribution map that encompasses both spectral and lifetime distributions. Irrespective of the probe energy, the LDD map exhibits distantly spaced distributions, with a notable sub-ps peak discernible in the time domain spanning the energy range of 3.4–4.2 eV. Since the LDD map serves as a quasi-continuous analogue of the DASs, the amplitudes observed at shorter lifetimes (e.g., <1 ps) exhibit a similar profile to DAS1. Meanwhile, the amplitudes at longer lifetimes (e.g., 10–100 ps) resemble DAS2, alongside the spectral traces observed during the long-term decay. This agreement further validates the reliability and reproducibility of the GLA and LDD fitting. Figure 4b showcases a lifetime trace taken at 3.8 eV, with the first peak (L1 in the figure) at ∼530 ± 106 fs, which represents the most probable time constant. Taking into account the variations in lifetimes observed at different detection energies around 3.8 eV (as shown in Figure S3, ∼420 ± 85 fs at 3.6 eV, ∼620 ± 124 fs at 3.7 eV, and ∼600 ± 124 fs at 3.9 eV, the discrepancy at ∼3.6 eV could result from fitting uncertainties and spectral overlap at this energy), we can conclude that the BER effect at this high-energy band exhibits a lifetime range of 400–600 fs, which aligns well with the BGR in the visible spectral region (see Figures 4c and S4, and the parameters in Table S2). Furthermore, as introduced previously in Figure 1c, the BGR-induced derivative-like spectral shape was generated through a Gaussian-like spectral peak red shift. In a reverse manner, we fit the DAS1 obtained from the UV probing using two subtracted Gaussian functions and determine a peak red shift of 150 ± 40 meV (see Figure 4c). This value aligns well with the observed fundamental BGR in the visible spectral region (see Figure S5 and Tables S3) and falls in the same level as the reported photoinduced BRG time scale,^47^ which occurs on the lifetime corresponding to DAS1 (∼400 fs).

## Conclusions

In summary, we carried out a broad-band TA spectroscopy spanning the visible and mid- to deep-UV probe ranges upon 3.1 eV excitation of MAPbBr_3_ thin films and combined it with advanced GLA and LDD analysis. The use of probe continua enables the investigation of excitations at various symmetry points within the BZ, offering a way to access them, which is otherwise not possible, even with momentum-resolved methods (e.g., ARPES or RIXS). Our results provide insights into the depth of kinetic information embedded within the experimental data and demonstrate that the transient gap shrinkage is identical at the *R* and *M* symmetry points. Since BGR/BER is caused by free carriers, its disappearance measures the time these free carriers decay to form neutral excitonic species that will decay radiatively. In this respect, the energy shift values reported here agree well with values reported for MAPbI_3_,^12^ MAPbI_2_Br,^31^ and CsPbBr_3_ quantum dots^7^ under low excitation fluence. A further extension of this work would be to monitor the response of (deeper) higher sub-bands of the (valence) conduction band, e.g., in the present case at transitions 2 and 4. Uncovering such higher-energy BERs, which has not previously been achieved, would shed light on the fundamental transient physical behaviors under nonequilibrium conditions in perovskite materials. These findings would also provide support for investigating underlying mechanisms^6,8,48^ such as ultrafast optical switching and unconventional superconductivity, behind carrier-induced bands renormalization.

## Supporting Information Available

Schematic of free carrier-induced effects on the electron–hole pair excitations; early time traces of the TA signals probed at 3.8 and 4.15 eV; lifetime traces of the LDD at the probe energy of 3.6, 3.7, and 3.9 eV; lifetime density distribution map within 100 ps; fitting of the DAS1 resulting from the visible probe signal; time traces of the rising TA signals probed at 3.4 and 3.8 eV; time evolution of the maximum peak at ∼3.6 eV fitted with two exponential functions; and time traces of the rising TA signals probed at 3.6 and 4.15 eV (PDF)
