# Supplementary material for: Band Gap Renormalization at Different Symmetry Points in Perovskites
Source: ACS Photonics. 2024 May 17;11(6):2273–81. doi: 10.1021/acsphotonics.4c00082 (PMC11191737; doi:10.1021/acsphotonics.4c00082)
Supplement: Supplementary file 1 — ph4c00082_si_001.pdf [file ph4c00082_si_001.pdf]

Supplementary Information

Band Gap Renormalization at Different Symmetry Points in Perovskites

Lijie Wang[1,2], Razan Nughays[2], Jun Yin[3], Chun-Hua Shih[4], Tzung-Fang Guo[4], Omar F. Mohammed[2,5*] & Majed Chergui[1,6*]

[1]*Laboratory of Ultrafast Spectroscopy, ISIC and Lausanne Centre for Ultrafast Science (LACUS), École Polytechnique Fédérale de Lausanne (EPFL), CH-1015 Lausanne, Switzerland.*

[2]*Advanced Membranes and Porous Materials Center (AMPM), Division of Physical Science and Engineering, King Abdullah University of Science and Technology (KAUST), Thuwal 23955-6900, Kingdom of Saudi Arabia.*

[3]*Department of Applied Physics, The Hong Kong Polytechnic University, Kowloon 999077, Hong Kong, P. R. China.*

[4]*Department of Photonics, National Cheng Kung University, Tainan 701, Taiwan ROC.*

[5]*KAUST Catalysis Center, Division of Physical Sciences and Engineering, King Abdullah University of Science and Technology (KAUST), Thuwal 23955-6900, Kingdom of Saudi Arabia.*

[6]*Elettra Sincrotrone Trieste, Strada Statale 14 - km 163,5, 34149 Basovizza, Trieste, Italy.*

Supplementary Note S1: The band assignments in the visible-to-deep-UV probe region

Figure 1b displays a typical absorption spectrum of $MAPbBr_3$ material, revealing a distinct excitonic feature appears at ~2.3 eV, accompanied by multiple absorption peaks at approximately 3.4 eV, 3.8 eV, and 4.45 eV. These features can be attributed to specific transitions based on the calculated band structure diagram presented in Figure 1c and they are annotated in the absorption spectrum. Specifically, they are identified as VB1 → CB1 at the R point (transition 1), corresponding to the BG transition in the visible spectral region; and VB3 → CB1 at the R point (transition 2), VB1 → CB1 at the M point (transition 3), and VB1 → CB1 at the X point (transition 4), respectively[1,2] in the mid- to deep-UV region.

The transient spectral traces of $MAPbBr_3$, probed in the visible and UV ranges, are depicted in Fig. 2a and 3a. The spectra exhibit distinct negative peaks located at approximately 2.35, 3.4 eV, 3.8 eV, and >4.3 eV. The energies of these bleaching (negative) signals align well with the ellipsometric measurements conducted within the same spectral range. Following a similar methodology used for the assignment of interband transitions in $MAPbI_3$[1,3], we determined the energy distances at each symmetry point in the Brillouin zone (BZ). Consequently, the ~2.35 eV peak is assigned to the transition from VB1 to CB1 at the R point, the ~3.4 eV peak is assigned to the transition from VB3 to CB1 at the R point, while the ~3.8 eV and ~4.45 eV peaks are assigned to transitions between VB1 and CB1 at the M and X points, respectively (Figure 1).

When excited below the energy gap at the M and X points, direct population at these high-symmetry points is not involved. Therefore, the transient signal at ~3.4 eV (R point) exhibits different sensitivity compared to the bleaches at ~3.8 eV (M point) and ~4.45 eV (X point). This distinction is apparent in the early time traces shown in Figure S6, where the transient signals at ~3.8 eV experiences a prompt rise, while the signal at ~3.4 eV rises more gradually due to the cooling of electrons towards

the bottom of CB at the M point. It is also worth to note that since our continuum probe covers the 3.3 to 4.3 eV range, which makes the fully detection of BER at transitions 2 and 4 unfeasible, and we therefore focus our study on transition 3 for the higher energy edge.

Supplementary Note S2: Decay associated spectra in the presence of time dependent spectral shifts

The analysis of time resolved data is of crucial importance for resolving the underlying physical quantities. A powerful and widely adopted tool for this purpose is global lifetime analysis (GLA)[4]. In GLA, a sum of exponentials with energy/wavelength dependent amplitudes is fitted, typically by least square routines, to the measured data. The outcome of such a procedure includes not only the exponential decay times but also decay associated spectra (DAS), which represent the energy/wavelength dependent amplitudes of the respective exponential contributions.

Inherent to the GLA-approach is the assumption that the time and energy/wavelength dependence of the data are separable. However, this becomes challenging in the presence of a time dependent spectral shift, as the basic assumption of separability of time and energy/wavelength dependence does not hold[5,6].

In the GLA-fitted TA map of perovskite materials, as shown in Figure 2 and 3, the spectral red-shift at the shortest timescales can yield an orthogonal component resembling a derivative-like shape. The DAS with a larger time constant reflects the transient signal due to photo-induced bleaching or absorption, while the DAS associated with shorter time constants, namely < 1 ps, parametrize the shift dynamics at the corresponding bands. Comparing the time constant of the DAS1 with the spectral shift time constants will elucidate whether this first process can be directly attributed to a physical process or is merely a distortion by the GLA[7]. In Figure S7, The overall shift at around ~3.6 eV can be related to an exponential shift with a time constant of 300 ± 100 fs, which is in good

agreement with the time constant of DAS1 (~360 fs). Additionally, the assumption of a preserved line-shape is fulfilled to a sufficient extent for the analysis presented here, and the evidence is that the full width at half maximum, for instance at around 3.6 eV, varies about 15% during the first 600 fs.

Nevertheless, it is essential to emphasize that the purpose of conducting a GLA of the time-energy maps of perovskite with a specifically 0-5 ps time window is to resolve information hidden due to spectral overlap, rather than to obtain kinetic parameters. The presence of a significant orthogonal component resembling a derivative-like shape (DAS1 in Figure 2d and 3e) serves as evidence that even in the high-energy mid-to-deep UV probe region, there is a spectral redshift similar to that of the BG transition immediately after photoexcitation near the M point in the BZ.

Table S1. Comparison of DAS lifetimes probed in the visible and UV spectral regions derived from GLA analysis.

	DAS1	DAS2
in the visible spectral region	0.55 ± 0.05 ps	35.87 ± 3.60 ps
in the UV spectral region	0.38 ± 0.06 ps	33.35 ± 3.50 ps

Table S2. Comparison of time constants at different energies resulting from LDD according to Eq. (3) at the fundamental BG at the R point and the higher BG at the M point.

At the fundamental gap (R point)	2.3 eV	2.4 eV	2.5 eV
	~530 ± 105 fs	~680 ± 136 fs	~510 ± 102 fs
At the M point gap	3.6 eV	3.7 eV	3.8 eV
	~420 ± 85 fs	~620 ± 124 fs	~530 ± 106 fs

Table S3. The extent of energy shift at the fundamental BG and at the M point.

Fundamental BG	M point gap
90 ± 30 meV	150 ± 40 meV

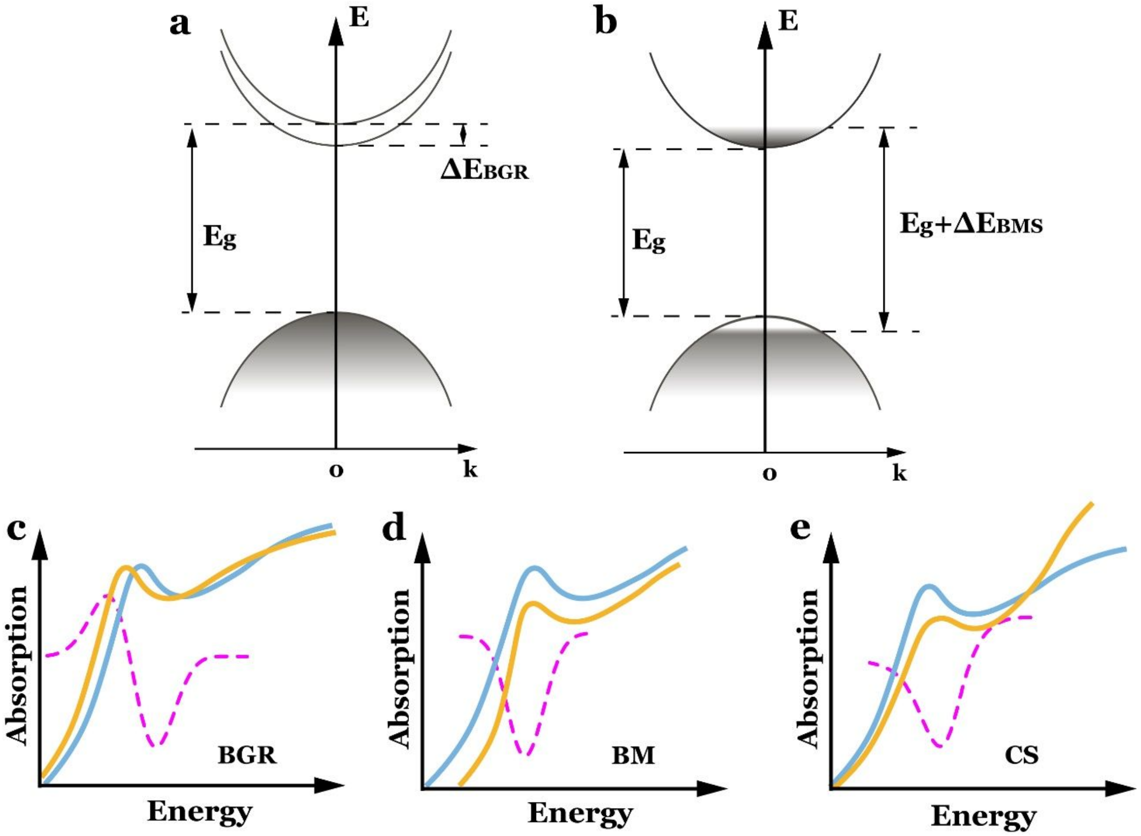

Figure S1. Schematic of free carrier-induced effects on the electron-hole pair excitations in the presence of: (a) bandgap renormalization. (b) Pauli Blocking of the lowest CB that causes a Burstein-Moss shift (ΔE_{BMS}). (c, d, and e) corresponding unexcited (blue) and excited (orange) absorption spectral responses and the difference spectral (dashed) at the edge states. BRG: bandgap renormalization. BM: Burstein-Moss effect. CS: Coulomb screening.

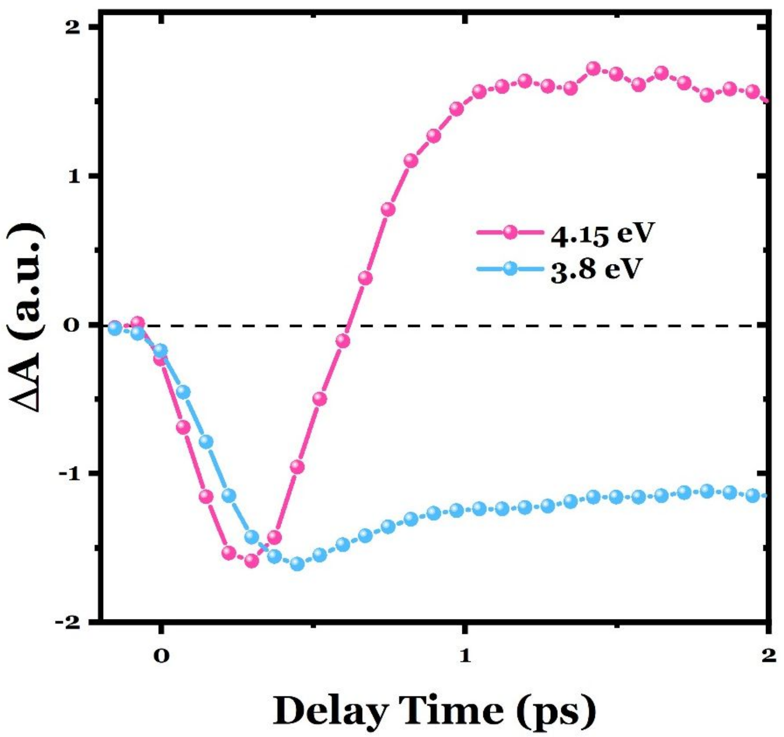

Figure S2. Early time traces of the TA signals probed at 3.8 and 4.15 eV, respectively. The traces are normalized at their minimum amplitudes.

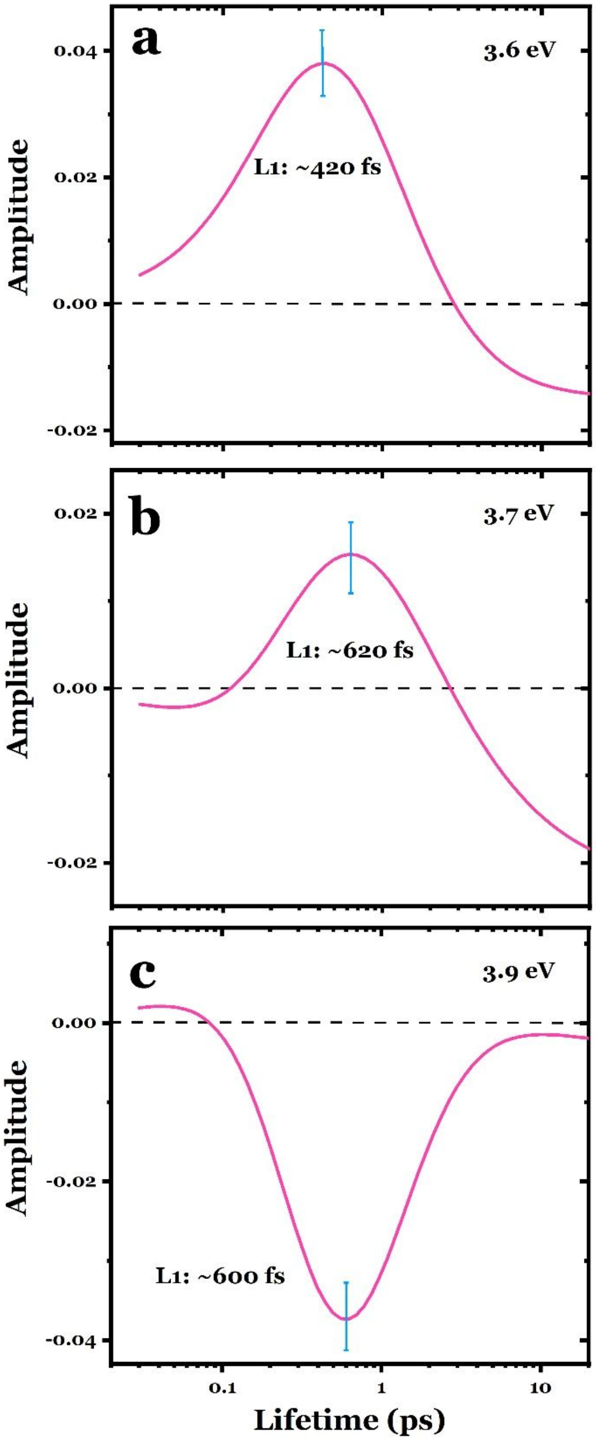

Figure S3. Lifetime traces of the LDD at the probe energy of a, 3.6 eV; b, 3.7 eV; c, 3.9 eV. A distinct peak can be resolved in the first 1 ps at different probed energies. The L1 peak position of at 3.6, 3.7, and 3.9 eV are ~420 ± 85, ~620 ± 124, and ~600 ± 124 fs, respectively.

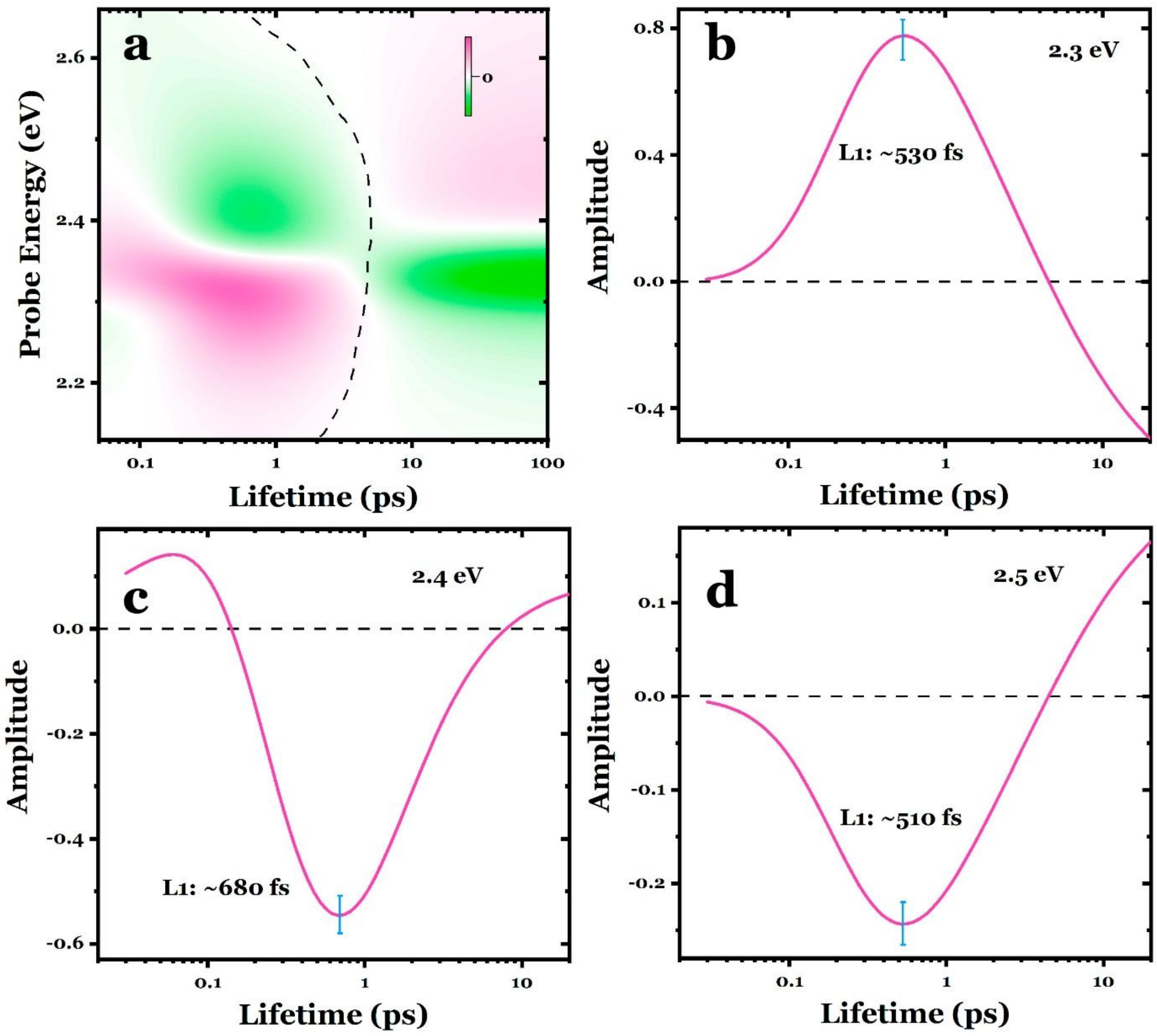

Figure S4. (a) Lifetime density distribution map within 100 ps, fitted to the ΔA data in Figure 2a. (b-d) Lifetime trace of the LDD at the probe energy of 2.3, 2.4, and 2.5 eV, a clear peak (L1) can be resolved in the first 1 ps, with the L1 at the amplitude maximum position of ~530 ± 106, ~680 ± 136, and ~510 ± 102 fs.

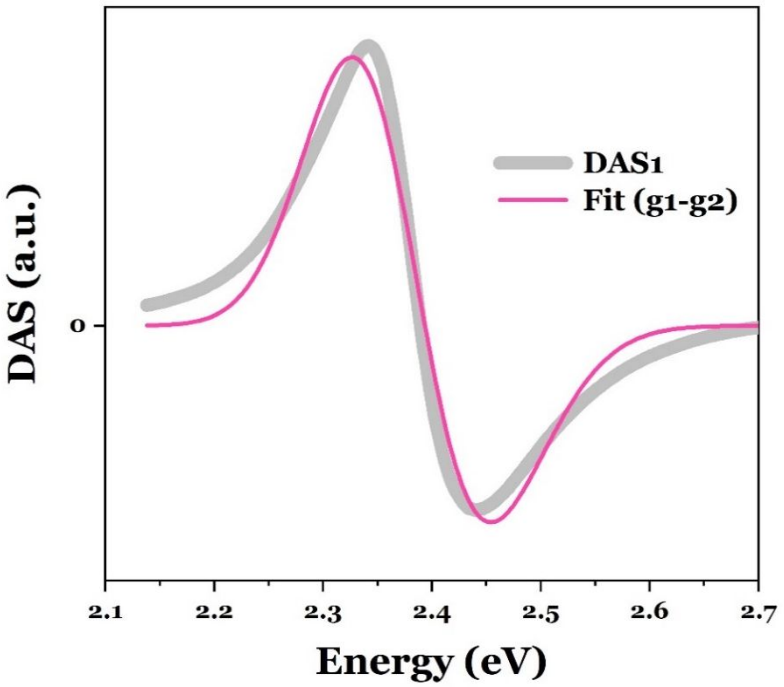

Figure S5. Fitting of the DAS1 resulting from the visible probe signal, using two Gaussian functions (Gaussian 1 minus Gaussian 2). A band shift of 90 ± 30 meV was obtained.

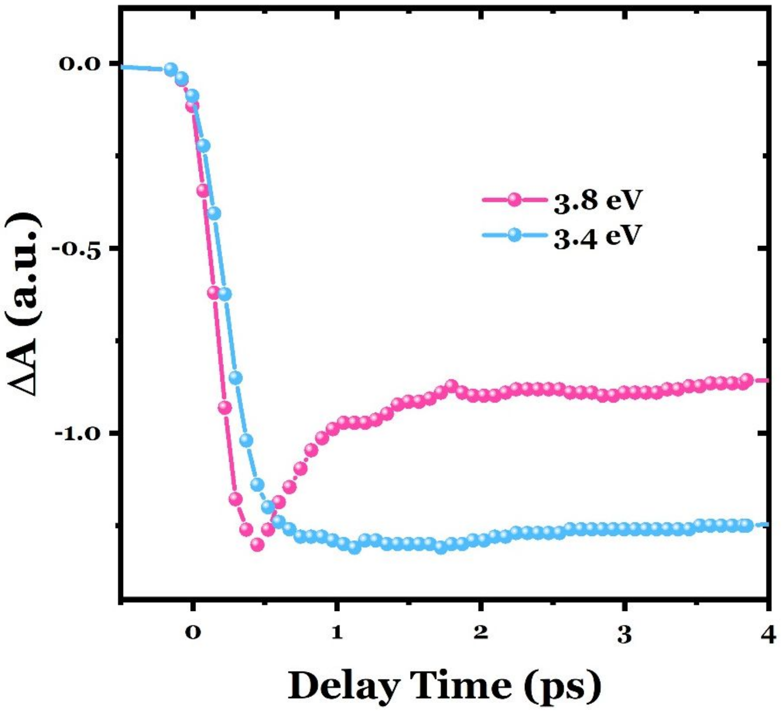

Figure S6. Time traces of the rising TA signals probed at 3.4 and 3.8 eV, respectively. The traces are zoomed in to the first 4 ps and normalized at their maximum amplitudes.

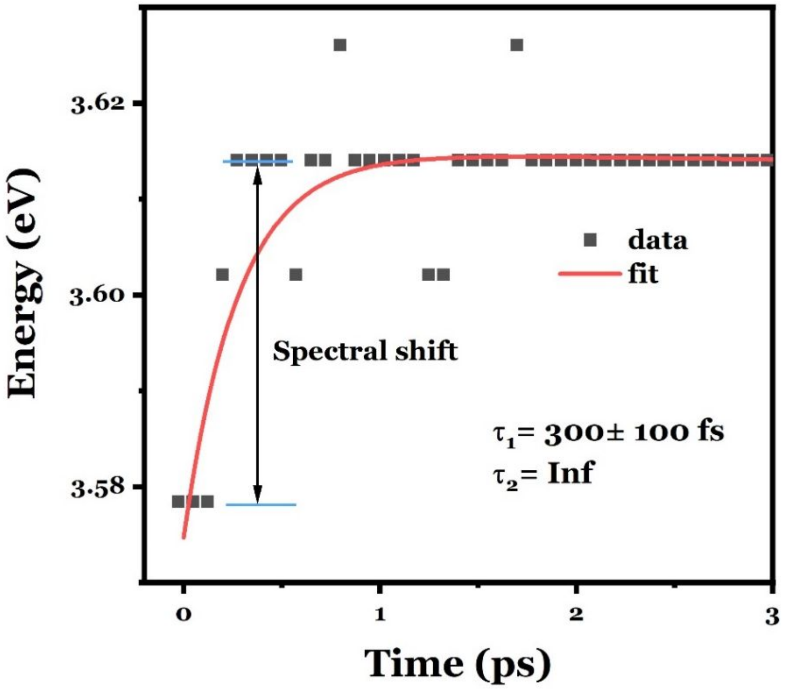

Figure S7. Time evolution of the maximum peak at ~3.6 eV fitted with two exponential functions. The resulting first time constant is 300 ± 100 fs.

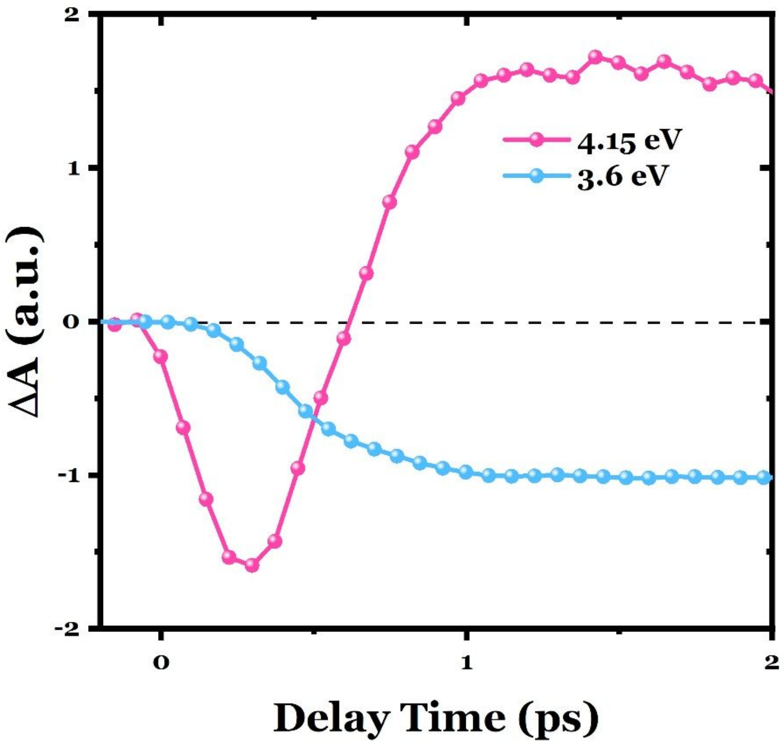

Figure S8. Time traces of the rising TA signals probed at 3.6 and 4.15 eV, respectively. Due to strong signal overlap, the positive signal at 3.6 eV is tiny, while at 4.15 eV, it even shows a negative signal at the beginning. However, the tiny positive signal at 3.6 eV rapidly decreases, while the signal at 4.15 eV increases with time.

References

(1) Leguy, A. M. A.; Azarhoosh, P.; Alonso, M. I.; Campoy-Quiles, M.; Weber, O. J.; Yao, J.; Bryant, D.; Weller, M. T.; Nelson, J.; Walsh, A.; van Schilfgaarde, M.; Barnes, P. R. F. Experimental and Theoretical Optical Properties of Methylammonium Lead Halide Perovskites. *Nanoscale* **2016**, *8* (12), 6317–6327.

(2) Mosconi, E.; Umari, P.; Angelis, F. D. Electronic and Optical Properties of $MAPbX_3$ Perovskites (X = I, Br, Cl): A Unified DFT and GW Theoretical Analysis. *Physical Chemistry Chemical Physics* **2016**, *18* (39), 27158–27164.

(3) Manser, J. S.; Kamat, P. V. Band Filling with Free Charge Carriers in Organometal Halide Perovskites. *Nature Photon* **2014**, *8* (9), 737–743.

(4) Ruckebusch, C.; Sliwa, M.; Pernot, P.; de Juan, A.; Tauler, R. Comprehensive Data Analysis of Femtosecond Transient Absorption Spectra: A Review. *Journal of Photochemistry and Photobiology C: Photochemistry Reviews* **2012**, *13* (1), 1–27.

(5) Fennel, F.; Lochbrunner, S. F\"orster-Mediated Spectral Diffusion in Disordered Organic Materials. *Phys. Rev. B* **2012**, *85* (9), 094203.

(6) Marciniak, H.; Teicher, M.; Scherf, U.; Trost, S.; Riedl, T.; Lehnhardt, M.; Rabe, T.; Kowalsky, W.; Lochbrunner, S. Photoexcitation Dynamics in Polyfluorene-Based Thin Films: Energy Transfer and Amplified Spontaneous Emission. *Phys. Rev. B* **2012**, *85* (21), 214204.

(7) On the Interpretation of Decay Associated Spectra in the Presence of Time Dependent Spectral Shifts. *Chemical Physics Letters* **2014**, *609*, 184–188.
